# Cooperative Assembly of a Protein-DNA Filament for Nonhomologous End Joining*

**DOI:** 10.1074/jbc.M113.464115

**Published:** 2013-04-25

**Authors:** Chun J. Tsai, Gilbert Chu

**Affiliations:** From the Departments of Medicine and Biochemistry, Stanford University School of Medicine, Stanford, California 94305

**Keywords:** DNA Recombination, DNA Repair, Immunology, Protein Complexes, Protein-DNA Interaction, Double-strand Break Repair, Nonhomologous End Joining, V(D)J Recombination

## Abstract

Nonhomologous end joining repairs DNA double-strand breaks created by ionizing radiation and V(D)J recombination. Ku, XRCC4/Ligase IV (XL), and XLF have a remarkable mismatched end (MEnd) ligase activity, particularly for ends with mismatched 3′ overhangs, but the mechanism has remained obscure. Here, we showed XL required Ku to bind DNA, whereas XLF required both Ku and XL to bind DNA. We detected cooperative assembly of one or two Ku molecules and up to five molecules each of XL and XLF into a Ku-XL-XLF-DNA (MEnd ligase-DNA) complex. XLF mutations that disrupted its interactions with XRCC4 or DNA also disrupted complex assembly and end joining. Together with published co-crystal structures of truncated XRCC4 and XLF proteins, our data with full-length Ku, XL, and XLF bound to DNA indicate assembly of a filament containing Ku plus alternating XL and XLF molecules. By contrast, in the absence of XLF, we detected cooperative assembly of up to six molecules each of Ku and XL into a Ku-XL-DNA complex, consistent with a filament containing alternating Ku and XL molecules. Despite a lower molecular mass, MEnd ligase-DNA had a lower electrophoretic mobility than Ku-XL-DNA. The anomalous difference in mobility and difference in XL to Ku molar ratio suggests that MEnd ligase-DNA has a distinct structure that successfully aligns mismatched DNA ends for ligation.

## Teaser

**Background:** Nonhomologous end-joining proteins can ligate DNA ends with mismatched overhangs.

**Results:** Ku facilitates the cooperative binding of multiple XRCC4/Ligase IV (XL) and XLF molecules to DNA.

**Conclusion:** The protein-DNA complex contains a filament of alternating XL and XLF molecules.

**Significance:** The filament may provide a mechanism for ligating mismatched DNA ends.

## Introduction

Nonhomologous end joining (NHEJ)^2^ repairs DNA double-strand breaks created by exogenous agents such as ionizing radiation, or by the endogenous mechanism for generating immunological diversity, V(D)J recombination (1, 2). NHEJ recognizes broken ends, brings them into juxtaposition, and joins the ends while optimizing the preservation of the DNA sequence. NHEJ even preserves 3′ overhanging DNA sequences created during V(D)J recombination by hairpin opening (P-nucleotide addition) or by terminal deoxynucleotidyl transferase activity (N-nucleotide addition). Thus, NHEJ has a mechanism for joining DNA ends with mismatched 3′ overhangs.

Core proteins for NHEJ include Ku, DNA-dependent protein kinase catalytic subunit (DNA-PKcs), XRCC4/Ligase IV (XL), and XRCC4-like factor (XLF, also known as Cernunnos). Ku, which consists of two subunits, Ku70 and Ku80, binds DNA ends with high affinity (3, 4) and then recruits DNA-PKcs to the ends (5). DNA-PKcs brings the ends together into a synaptic complex and then undergoes activation of its kinase domain to facilitate later steps in the joining reaction (6). XL ligates the ends (7).

The *XLF* gene was identified from patients with a rare autosomal recessive disorder of microcephaly, radiosensitivity, and immunodeficiency due to defective V(D)J recombination (8). Knock-out of *XLF* in mouse embryonic stem cells leads to defective V(D)J recombination (9). Surprisingly, knock-out of *XLF* in the whole mouse has little effect on immune function because of lymphocyte-specific compensation by the ATM kinase (10, 11). For unknown reasons, compensation by ATM does not occur in humans.

XLF is weakly homologous to and interacts with XRCC4 (12). Each protein is a homodimer with an N-terminal globular head domain and a C-terminal α-helical tail domain (13, 14). Biochemical analysis of mutated proteins shows that XRCC4 and XLF interact via their head domains (13, 15). Co-crystals of C-terminal truncated XRCC4 and XLF show head-to-head interactions in a helical filament of alternating XRCC4 and XLF molecules (16–19). Small angle x-ray scattering in solution of full-length XRCC4 and XLF was consistent with an alternating structure (20). However, the co-crystal structures lacked DNA, Ku, and Ligase IV.

Ku and XL efficiently ligate ends with cohesive overhangs. The addition of XLF stimulates a latent mismatched end (MEnd) ligase activity, particularly for ends with mismatched 3′ overhangs (21, 22). The molecular mechanism for this remarkable MEnd ligase activity has remained obscure. Here, we developed an electrophoretic mobility shift assay (EMSA) to study the binding of full-length Ku, XL, and XLF to DNA. Ku bound to DNA recruited XL and XLF, leading to the cooperative formation of progressively larger complexes.

We also developed a two-step gel elution procedure of EMSA followed by SDS-PAGE to show that Ku and XL formed a Ku-XL-DNA complex, which contained up to six molecules each of Ku and XL. By contrast, Ku, XL, and XLF formed a Ku-XL-XLF-DNA complex (hereafter designated the MEnd ligase-DNA complex), which contained one or two Ku molecules and three to five molecules each of XL and XLF. Surprisingly, a 1.1-MDa MEnd ligase-DNA complex had a lower electrophoretic mobility than a 2.2-MDa Ku-XL-DNA complex. These results show that the MEnd ligase-DNA complex forms a filament containing alternating XL and XLF molecules. The filament may facilitate alignment of DNA ends so that mismatched 3′ overhangs can undergo ligation by one of several Ligase IV molecules arrayed along the DNA.

## EXPERIMENTAL PROCEDURES

### 

#### 

##### Protein Expression and Purification

Ku70/Ku80, XRCC4/Ligase IV, and XLF were expressed and purified to homogeneity as described previously (22). The ybbR-tagged XLF consisted of an N-terminal ybbR tag (23) added to XLF-V5-His_6_.

##### Conjugation of Tetramethylrhodamine (TAMRA) to ybbR-tagged XLF and Purification

Purified ybbR-tagged XLF was labeled with the fluorophore TAMRA using TAMRA-CoA (a generous gift from Dr. Kierstin Schmidt) by Sfp phosphopantetheinyl transferase (23), purified on a 1-ml Hi-Trap heparin column (GE Healthcare) using a linear salt gradient of 150–1000 mm NaCl in Na-PO_4_, flash-frozen, and stored at −80 °C.

##### Preparation and Labeling of DNA Probes

**FIGURE 1.**
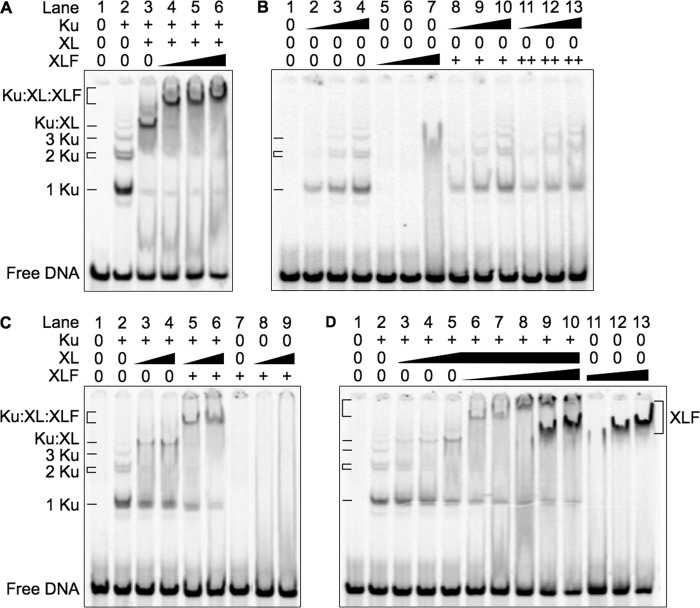
**Ku, XL and XLF bind to DNA as a large protein-DNA complex.** Binding reactions contained a 5′ ^33^P-labeled 298-bp DNA probe (1 nm). *A*, Ku, XL, and XLF bind sequentially to DNA to form the MEnd ligase-DNA complex. Concentrations of Ku and XL, when present, were 2.4 and 9.6 nm, respectively. XLF was added to 4.8 nm (*lane 4*), 9.6 nm (*lane 5*), and 19.2 nm (*lane 6*). *B*, XLF fails to form a stable complex with Ku in the absence of XL. Ku was added to 0.3 nm (*lanes 2*, *8*, and *11*), 0.6 nm (*lanes 3*, *9*, and *12*), and 1.2 nm (*lanes 4*, *10*, and *13*). XLF was added to 2.4 nm (*lanes 5*, *8*, *9*, and *10*), 4.8 nm (*lanes 6*, *11*, *12*, and *13*), and 9.6 nm (*lane 7*). *C*, recruitment of XLF and XL to the MEnd ligase-DNA complex requires Ku. Concentrations of Ku and XLF, when present, were 1.2 and 9.6 nm, respectively. XL was added to 2.4 nm (*lanes 3*, *5*, and *8*) and 4.8 nm (*lanes 4*, *6*, and *9*). *D*, increasing XLF results in progressively larger MEnd ligase-DNA complexes. The concentration of Ku, when present, was 1.2 nm. XL was added to 1.2 nm (*lane 3*), 2.4 nm (*lane 4*), and 4.8 nm (*lanes 5–10*). XLF was added to 2.4 nm (*lane 6*), 4.8 nm (*lane 7*), 9.6 nm (*lanes 8* and *11*), 19.2 nm (*lanes 9* and *12*), and 38.4 nm (*lanes 10* and *13*).

**FIGURE 2.**
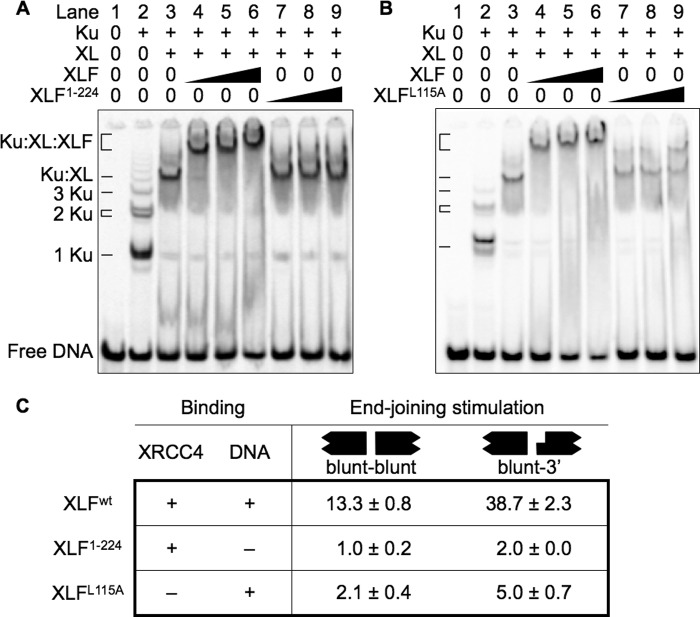
**Mutant XLF proteins defective in MEnd ligase-DNA complex formation are also defective in stimulating end joining.** Binding reactions contained a 5′ ^33^P-labeled 298-bp DNA probe (1 nm). *A*, XLF^1–224^ fails to support formation of the MEnd ligase-DNA complex. Concentrations of Ku and XL, when present, were 2.4 and 9.6 nm, respectively. XLF was added to 4.8 nm (*lanes 4* and *7*), 9.6 nm (*lanes 5* and *8*), and 19.2 nm (*lanes 6* and *9*). *B*, XLF^L115A^ fails to support stable formation of the MEnd ligase-DNA complex. Concentrations of Ku and XL, when present, were 1.2 and 4.8 nm, respectively. XLF was added to 2.4 nm (*lanes 4* and *7*), 4.8 nm (*lanes 5* and *8*), and 9.6 nm (*lanes 6* and *9*). *C*, XLF^1–224^ and XLF^L115A^ mutant proteins are defective in stimulating end joining. End-joining stimulation was the -fold change in joining efficiency induced by addition of XLF to Ku and XL. The 800-bp DNA substrates contained blunt-blunt (EcoRV-EcoRV) ends, or blunt-3′ overhanging (EcoRV-KpnI) ends. Uncertainties were estimated from duplicate experiments. The XLF^1–224^ and XLF^L115A^ mutant proteins fail to bind to DNA and XRCC4, respectively (13).

**FIGURE 3.**
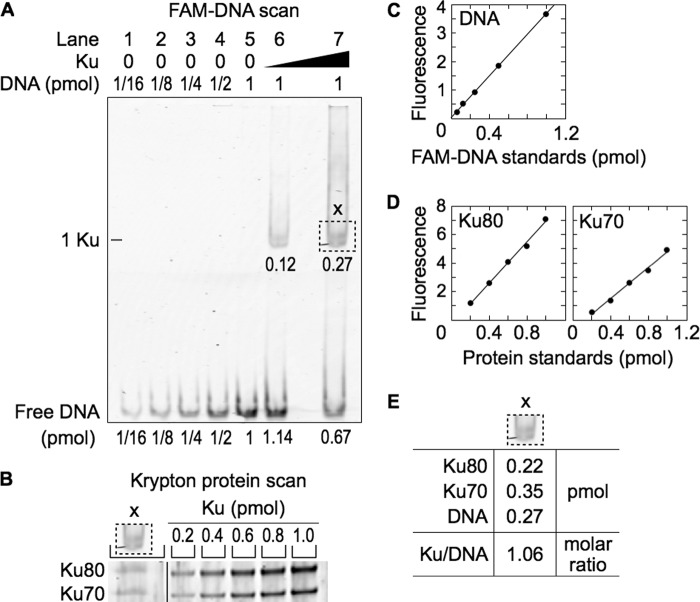
**Ku-DNA complex validates the two-step gel elution procedure.** *A*, EMSA for the protein-DNA complex. Ku (40 and 300 nm) was added to 6-FAM-labeled 180-bp DNA (100 nm) and resolved by EMSA. *B*, SDS-PAGE of the Ku-DNA complex. The *dotted box* (*x*) was excised from the EMSA gel, denatured and resolved by SDS-PAGE. The *vertical line* in the gel indicates removal of superfluous lanes. *C*, standard curve for DNA quantification. *D*, standard curves for protein quantification. *E*, DNA and protein quantification of the protein-DNA complexes.

**FIGURE 4.**
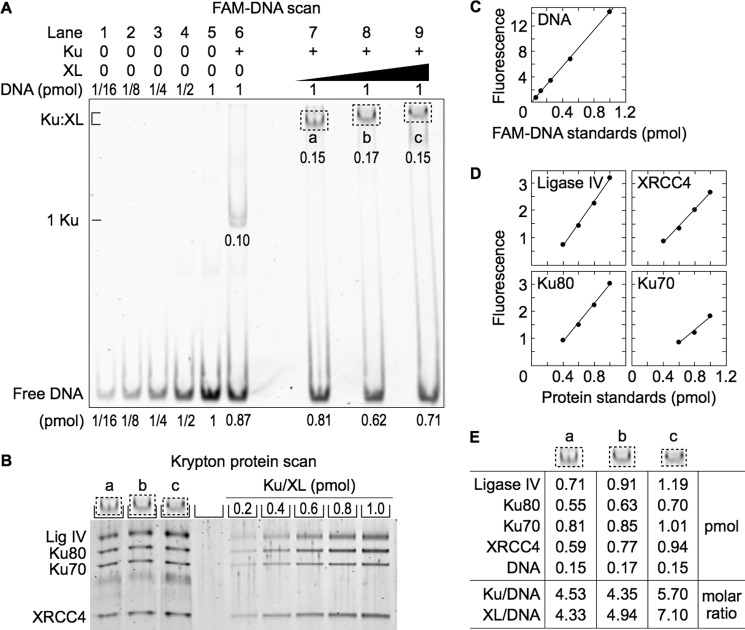
**Ku-XL-DNA complex contains approximately equal numbers of Ku and XL molecules.** *A*, EMSA for the protein-DNA complex. Ku (65 nm when present) and XL (50, 100, and 200 nm, *lanes 7–9*) were added to 6-FAM-labeled 180-bp DNA (100 nm) and resolved by EMSA. *B*, SDS-PAGE of the Ku-XL-DNA complex. The *dotted boxes* (*a*, *b*, and *c*) were excised from the EMSA gel, denatured, and resolved by SDS-PAGE. *C*, standard curve for DNA quantification. *D*, standard curves for protein quantification. *E*, DNA and protein quantification of the protein-DNA complexes.

**FIGURE 5.**
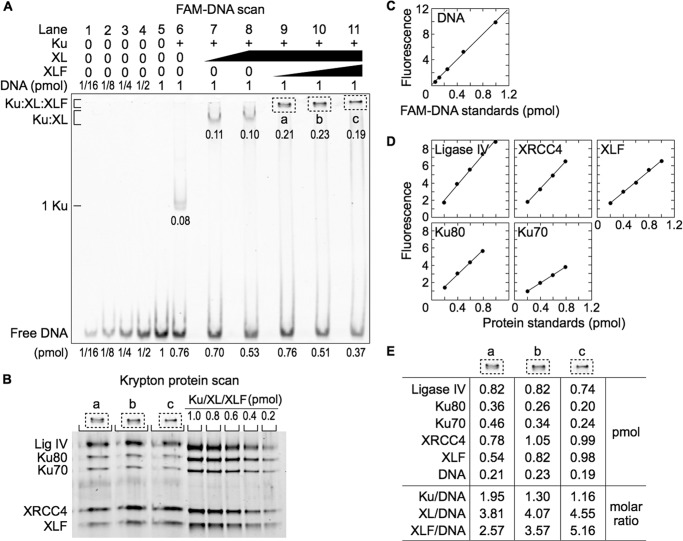
**MEnd ligase-DNA complex contains approximately equal numbers of XL and XLF molecules.** *A*, EMSA for the protein-DNA complex. Ku (40 nm when present), XL (100 nm, *lane 7*, and 200 nm, *lanes 8–11*), and XLF (50, 100, and 200 nm, *lanes 9–11*) were added to 6-FAM-labeled 180-bp DNA (100 nm) and resolved by EMSA. *B*, SDS-PAGE of the MEnd ligase-DNA complex. The *dotted boxes* (*a*, *b*, and *c*) were excised from the EMSA gel, denatured, and resolved by SDS-PAGE. *C*, standard curve for DNA quantification. *D*, standard curves for protein quantification. *E*, DNA and protein quantification of the protein-DNA complexes.

**FIGURE 6.**
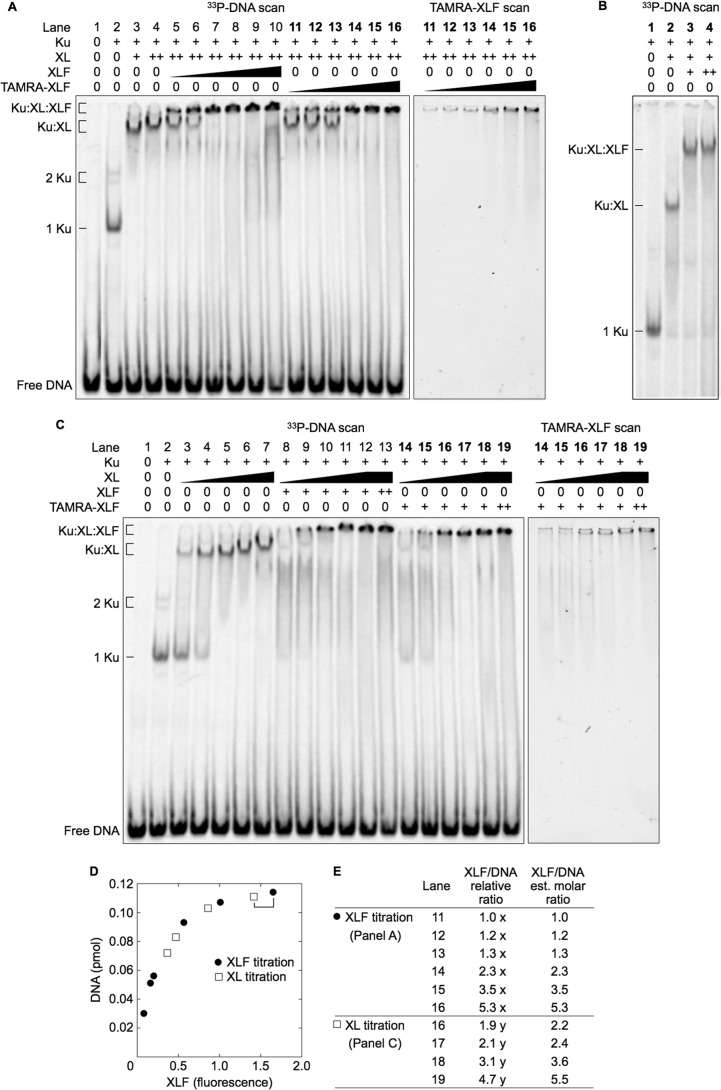
**MEnd ligase-DNA complex can contain one to five XLF molecules.** *A* and *C*, Ku (20 nm when present) and different concentrations of XL and XLF were added to ^33^P-labeled 180-bp DNA probe (50 nm) and resolved by EMSA. *A*, increasing XLF forms larger MEnd ligase-DNA complexes. XL concentrations were 50 nm (*lane 3*) or 100 nm (*lanes 4–16*). XLF and XLF-TAMRA were added to 3, 6, 12.5, 25, 50, or 100 nm (*lanes 5–16*). *B*, the MEnd ligase-DNA complex has a distinct mobility. Ku (0.6 nm), XL (2.6 nm), and XLF (1.3 or 2.6 nm) were added to a ^33^P-labeled 180-bp DNA probe (1.3 nm) and resolved by EMSA. Protein and DNA concentrations were decreased proportionally to increase the electrophoretic mobility of the complex. *C*, increasing XL forms larger MEnd ligase-DNA complexes. XLF concentrations were 50 nm (*lanes 8–12*) or 100 nm (*lane 13*). TAMRA-XLF concentrations were 50 nm (*lanes 14–18*) or 100 nm (*lane 19*). XL concentrations were 6, 12.5, 25, 50, or 100 nm (*lanes 3–7*, *lanes 8–12*, and *lanes 14–18*). For *lanes 13* and 19, the XL concentration was 100 nm. *D*, DNA amount was plotted against TAMRA-XLF fluorescence intensity. Data are results from scanning gels in *A* and *C* for [^33^P]DNA and TAMRA-XLF. *E*, the relative ratios of XLF to DNA are expressed as multiples of unknown variables *x* and *y* for the gels in *A* and *C*, respectively. The amounts of Ku, XL, XLF, and DNA in the binding reactions for *A* were present in the same molar ratios, at lower absolute concentrations as the binding reactions for Fig. 5. Therefore, we set *x* = 1, its minimum value. Because the binding reactions in *A*, *lanes 15* and *16*, and *C*, *lanes 18* and *19*, were identical, setting *y* = 1.17 normalized the fluorescence in *C*, *lane 19*, to the fluorescence in *A*, *lane 16*.

To study the formation of protein-DNA complexes, blunt-ended 298-bp and 180-bp DNA probes were prepared by PCR amplification of the bacterial *amp*^R^ gene and purified with a QIAquick PCR purification kit (Qiagen). For experiments in Figs. 1, 2, and 6, DNA probes were labeled with ^33^P using T4 polynucleotide kinase (New England Biolabs) and purified again with nucleotide removal kit (Qiagen). For experiments in Figs. 3–5, the 180-bp DNA probe was fluorescently labeled by PCR amplification using a forward primer with 6-carboxyfluoroscein (6-FAM) conjugated to the 5′ end (Elim Biopharm).

##### EMSA

Proteins were incubated with a labeled DNA probe for 30 min at 37 °C in 10 μl of end-joining buffer, which consists of 25 mm Tris (pH 7.5), 75 mm NaCl, 72.5 mm KCl, 5 mm MgCl_2_, 0.1 mm EDTA, 50 μg/ml BSA, 0.05% Triton X-100, 2 mm DTT, 5% glycerol, and 5% (w/v) polyethylene glycol (molecular mass >8000 kDa). Reaction products were resolved by electrophoresis at 10 V/cm in 0.5× TBE buffer containing 45 mm Tris borate (pH 8.0) and 1 mm EDTA. Initial experiments used precast commercial 5% polyacrylamide gels (Bio-Rad) (Figs. 1 and 2). Subsequent lots of the precast gels failed to stabilize the megadalton protein-DNA complexes. Therefore, we cast our own gels for other experiments and obtained highly reproducible results, using 6.5% polyacrylamide (74:1, w/w, acrylamide to bisacrylamide) (Fig. 4) or 6% polyacrylamide (59:1, w/w, acrylamide to bisacrylamide) (Figs. 3, 5, and 6) in 0.5× TBE buffer.

##### Measurement of Protein-DNA Stoichiometry

Stoichiometry was measured in a two-step gel elution procedure (Figs. 3–5) In the first step, purified proteins were incubated with DNA, and the protein-DNA complexes were resolved by EMSA. In the second step, the protein-DNA complexes were cut from the EMSA gel, and the proteins were analyzed by SDS-PAGE.

For the first step, the DNA probe was labeled with 6-FAM. To limit the size of complexes on the DNA, the DNA probe was 180 bp, which was shorter than the 298-bp DNA probe used in initial EMSA experiments in Figs. 1 and 2. Proteins were incubated with 6-FAM-DNA. To measure the DNA in the protein-DNA complex, protein-DNA complexes were resolved by EMSA in parallel with dilutions of unbound 6-FAM-DNA (1/16, 1/8, 1/4, 1/2, and 1 pmol). The gel was scanned for 6-FAM fluorescence (Typhoon imager; GE Healthcare) to generate plots of background-subtracted fluorescence intensities *versus* known amounts of DNA. The plots were highly linear (Figs. 3*C*, 4*C*, and 5*C*) with Pearson's correlation coefficients *r*^2^ > 0.99. The plot of DNA standards was used to estimate the amount of DNA in the protein-DNA complex.

For the second step, the DNA in the EMSA gel was stained with SYBR Gold (Invitrogen), allowing the protein-DNA complexes to be visualized on a UV transilluminator, and cut from the gel with a clean scalpel. However, for analysis of the MEnd ligase-DNA complex, SYBR Gold staining of the DNA led to loss of XLF protein. Therefore, we used the fluorescence image of the gel to cut the MEnd ligase-DNA complex from the gel (Fig. 5).

To denature the proteins in the protein-DNA complex, the gel slice was incubated in lithium dodecyl sulfate-PAGE sample buffer (Invitrogen) and heated for 30 min at 85 °C. To measure the protein in the protein-DNA complex, the gel slice together with the incubation sample buffer was loaded into a well, and dilutions of unbound proteins (0.2, 0.4, 0.6, 0.8, and 1 pmol) were loaded into parallel wells. The proteins were resolved by SDS-PAGE in a denaturing 4–12% gradient polyacrylamide gel (Invitrogen)

The gel was stained with Krypton protein stain (Pierce) and scanned to generate plots of background-subtracted fluorescence intensities *versus* known amounts of protein. Krypton protein stain provided better sensitivity than Coomassie Blue and a wider quantitative range than silver staining. The plots were highly linear (Figs. 3*D*, 4*D*, and 5*D*), with *r*^2^ > 0.97. The plots of protein standards were used to determine the amount of protein in the gel slice containing protein-DNA complex.

Replicate experiments and titrations provided estimates for the reliability of each component of the two-step gel elution procedure. DNA quantification was highly reliable with an uncertainty of 1%. Protein quantification was reliable to an uncertainty of 10%. Protein to DNA molar ratios, based on three or more replicate experiments with the full two-step gel elution procedure, revealed an overall uncertainty of 20%.

##### In Vitro End-joining Reactions

End-joining reactions were performed in end-joining buffer as described previously (22). The 800-bp linear DNA substrates with EcoRV-EcoRV or EcoRV-KpnI ends were prepared for end joining as described previously (22). Proteins were preincubated with DNA for 10 min at 25 °C. To initiate ligation, MgCl_2_ and ATP were added to final concentrations of 5 and 0.1 mm, respectively. Unless otherwise noted in the figure legends, reactions were performed in 20 μl with 1 nm DNA, 5 nm Ku, 2 nm XL, and 2.5 nm XLF at 37 °C for 30 min, and stopped by adding 2 μl of 0.5 m EDTA (pH 8.0). DNA was purified using QIAquick PCR purification kit (Qiagen) and joining efficiencies measured by quantitative PCR as described previously (24).

## RESULTS

### 

#### 

##### Ku, XL, and XLF Bind Sequentially to DNA with Cooperative Addition of XL and XLF

To understand how Ku, XL, and XLF form a complex on DNA, we used an EMSA to resolve protein-DNA complexes from binding reactions (see “Experimental Procedures”). Ku binding to DNA was distributive because it produced a series of protein-DNA complexes containing one, two, and three or more Ku molecules (Fig. 1*A*, *lane 2*). When XL was added, the Ku-DNA complexes were replaced by a lower mobility Ku-XL-DNA complex (Fig. 1*A*, *lane 3*).

When XLF was added, an even lower mobility MEnd ligase-DNA complex replaced the Ku-XL-DNA complex. Increasing concentrations of XLF formed progressively lower mobility complexes (Fig. 1*A*, *lanes 4–6*). Ku and XLF failed to form a well defined protein-DNA complex in the absence of XL, although a faint smear between the 1 Ku and 2 Ku bands suggested formation of an unstable Ku-XLF-DNA complex (Fig. 1*B*, *lanes 8–13*). This was consistent with results in living cells where recruitment of XLF by Ku to double-strand breaks is unstable in the absence of XRCC4 (25). With Ku absent, XL and XLF failed to form a protein-DNA complex (Fig. 1*C*, *lanes 8–9*). With Ku and XL absent, XLF-DNA complexes formed only at high XLF concentrations (Fig. 1*D*, *lanes 9–13*). Thus, Ku binding to DNA facilitated recruitment of XL and XLF.

At lower concentrations of Ku (1.2 nm) and XL (4.8 nm), addition of XLF over a broad range of concentrations (2.4–38.4 nm) formed complexes with a broad range of mobility (Fig. 1*D*, *lanes 6–10*). Each XLF concentration formed a complex with a characteristic mobility rather than a series of complexes with different mobilities. Whereas binding of Ku to DNA was distributive, binding of Ku, XL, and XLF to DNA was cooperative and suggested binding of multiple XLF molecules.

##### XLF Mutations That Disrupt the MEnd Ligase-DNA Complex Also Disrupt End Ligation

To determine whether XLF must bind both DNA and XL to form a MEnd ligase-DNA complex, we studied two XLF mutant proteins, XLF^1–224^ and XLF^L115A^. XLF^1–224^, which deletes C-terminal amino acids 225–299 and disrupts binding to DNA (13), failed to form a MEnd ligase-DNA complex and failed to stimulate blunt-to-blunt and blunt-to-3′ overhang end ligation (Fig. 2, *A*, *lanes 7–9*, and *C*). XLF^L115A^, which disrupts binding to XRCC4 (13), formed a barely detectable MEnd ligase-DNA complex, consistent with its weak stimulation of blunt-to-blunt and blunt-to-3′ overhang end ligation (Fig. 2, *B*, *lanes 7–9*, and *C*). Thus, defects in formation of the MEnd ligase-DNA complex correlated with defects in blunt end and mismatched end ligation.

##### A Two-step Gel Elution Procedure Measures the Stoichiometry of Protein-DNA Complexes

To determine the stoichiometry of the protein-DNA complexes, we developed a two-step gel elution procedure (“Experimental Procedures”). The first step resolved the protein-DNA complex by EMSA. The second step excised the complex from the EMSA gel and resolved the DNA-bound proteins by SDS-PAGE.

To validate the procedure, we performed a pilot experiment by incubating Ku with DNA and using an EMSA to resolve the protein-DNA complex containing a single Ku molecule (Fig. 3*A*, *lanes 6* and *7*). In the second step, we excised the Ku-DNA complex from the EMSA gel (marked by the *dotted lines*) and used SDS-PAGE to resolve the proteins contained in the gel slice (Fig. 3*B*).

Ku is an obligate heterodimer of Ku70 and Ku80. Because the measured amounts of Ku70 and Ku80 in the gel slice were 0.35 pmol and 0.22 pmol, respectively, we estimated the amount of Ku to be the average of the two values, 0.29 pmol. This was nearly identical to the amount of DNA, 0.27 pmol, and consistent with the predicted 1:1 molar ratio of Ku to DNA (Fig. 3*E*).

##### Ku and XL Form a Ku-XL-DNA Complex Containing Multiple Ku and XL Molecules

Addition of increasing concentrations of XL to Ku and DNA formed a Ku-XL-DNA complex of progressively lower mobility without evidence of multiple bands (Fig. 4*A*, *lanes 7–9*). Thus, binding of Ku and XL to DNA was cooperative. We scanned the gel for fluorescence and quantified the 6-FAM-DNA in the Ku-XL-DNA complex (Fig. 4, *A* and *C*). Proteins in Ku-XL-DNA gel slices were resolved by SDS-PAGE and quantified by Krypton protein stain using dilutions of unbound Ku and XL (Fig. 4, *B–E*).

As in the pilot experiment, the amount of Ku70 was somewhat higher than the amount of Ku80. XL was purified as a stable protein complex of XRCC4 and Ligase IV, and the expected molar ratio of XRCC4 dimer to Ligase IV was 1:1. The measured XRCC4 to Ligase IV molar ratios were somewhat less than 1:1. These deviations indicated an uncertainty of 10% in protein quantification.

In the Ku-XL-DNA complex, each DNA molecule bound four to seven molecules of Ku and XL (Fig. 4*E*). The presence of multiple Ku molecules in the Ku-XL-DNA complex was surprising because in the absence of XL, the Ku concentration used in the binding reaction generated mostly a one Ku-DNA complex (Fig. 4*A*, *lane 6*). For successive 2-fold increases in XL concentration, the XL to Ku molar ratio in the Ku-XL-DNA complex increased slowly from 1.0 to 1.1 to 1.2. Thus, Ku binding to DNA recruits XL (Fig. 1), and XL recruits Ku. This suggests that the Ku-XL-DNA complex consists of a filament of alternating Ku and XL molecules.

##### Ku, XL, and XLF Form a MEnd Ligase-DNA Complex Containing Multiple XL and XLF Molecules

To measure the stoichiometry of the MEnd ligase-DNA complexes, we adjusted EMSA gel matrix to generate sharper bands. This decreased the uncertainty in protein to DNA molar ratio measurements, which require matching the gel region scanned for DNA and the gel slice analyzed for protein. Because the new gel matrix might affect recovery of proteins from the EMSA gel slice, we checked the protein-to-protein molar ratio in the Ku-XL-DNA gel slices from the new gel matrix (Fig. 5*A*, *lanes 7* and *8*) and confirmed that it remained near 1:1 (data not shown).

Addition of increasing concentrations of XLF to Ku, XL, and DNA formed MEnd ligase-DNA complexes of progressively lower mobility (Fig. 5*A*, *lanes 9–11*). For successive 2-fold increases in XLF concentration, the number of XLF molecules increased from 2.6 to 3.6 to 5.1, and the number of XL molecules increased from 3.8 to 4.1 to 4.6 (Fig. 5, *B–E*). Thus, Ku and XL binding to DNA recruits XLF (Fig. 1), and XLF binding recruits XL. This suggests that the MEnd ligase-DNA complex contains a filament of alternating XL and XLF molecules.

The MEnd ligase-DNA complex contained fewer Ku molecules with increased XL and XLF binding, declining from 2.0 to 1.3 to 1.1. This result was unexpected because Ku has a higher affinity for DNA than either XL or XLF (Fig. 1, *B* and *C*) and because the Ku-XL-DNA complex contained more Ku molecules with increased XL binding. Thus, assembly of progressively larger MEnd ligase-DNA complexes resulted in an increasing XL to Ku molar ratio, whereas by contrast, the assembly of progressively larger Ku-XL-DNA complexes maintained an XL to Ku molar ratio close to 1:1.

##### The MEnd Ligase-DNA Complex Forms More Efficiently Than the Ku-XL-DNA Complex

Although the two-step gel elution procedure was designed to ensure its accuracy, we wanted a direct and more sensitive method for estimating the number of XLF molecules in the MEnd ligase-DNA complex. Therefore, we labeled XLF with TAMRA, which increased XLF detection sensitivity by 10-fold. This eliminated the gel excision step by allowing *in situ* quantification of XLF in the EMSA gel. The DNA probe was labeled with ^33^P rather than 6-FAM to avoid interference with the TAMRA signal.

To confirm that TAMRA labeling preserved the MEnd ligase-DNA complex, we added unlabeled XLF or TAMRA-labeled XLF in increasing concentrations to Ku, XL, and DNA and resolved the complexes by EMSA. XLF and TAMRA-XLF generated similar patterns of bands with two ranges of mobility (Fig. 6*A*, *lanes 5–16*). Only the lower mobility bands contained TAMRA (Fig. 6*A*, *right panel*), confirming that XLF and TAMRA-XLF formed similar MEnd ligase-DNA complexes.

Close inspection of the gel showed that the lower mobility bands did migrate into the gel, particularly for lower concentrations of XLF (Fig. 6*A*, *lanes 5–7* and *11–13*). To confirm that the band represented a well defined protein-DNA complex and not protein-DNA aggregates, we allowed the free DNA to migrate off the bottom of the gel (Fig. 6*B*).

An XLF concentration of only 3 nm was sufficient to form the same amount of MEnd ligase-DNA and Ku-XL-DNA complexes despite much higher concentrations of XL (100 nm) and of Ku (20 nm) (Fig. 6*A*, *lane 5*). Furthermore, an XLF concentration of only 13 nm resulted in disappearance of the Ku-XL-DNA complex (Fig. 6*A*, *lane 7*). TAMRA-XLF also formed a MEnd ligase-DNA complex and suppressed the Ku-XL-DNA complex, albeit to a slightly lesser degree. This is probably due to a small decrease in DNA binding because the smear of radioactivity from direct binding of XLF to DNA was less prominent for TAMRA-XLF (Fig. 6*A*, compare *lanes 10* and *16*). Nevertheless, for both XLF and TAMRA-XLF, the MEnd ligase-DNA complex formed much more readily than the Ku-XL-DNA complex.

To study the MEnd ligase-DNA complex further, we added increasing concentrations of XL to Ku and unlabeled XLF, or to Ku and TAMRA-labeled XLF. The results for unlabeled and TAMRA-labeled XLF were similar, showing that TAMRA labeling preserves the MEnd ligase-DNA complex (Fig. 6*C*, compare *lanes 8–13* with *lanes 14–19*). XL at 25 nm formed a MEnd ligase-DNA complex with minimal formation of Ku-XL-DNA or Ku-DNA complexes (Fig. 6*C*, *lanes 10* and 16). This confirmed the result in Fig. 6*A*, showing that the MEnd ligase-DNA complex forms more readily than the Ku-XL-DNA complex. Moreover, increasing XL concentrations recruited additional XLF molecules.

To estimate the molar ratio of XLF to DNA, we performed *in situ* measurements of the EMSA gels for TAMRA-XLF and ^33^P-labeled DNA (Fig. 6, *A*, *C*, and *D*). These measurements showed that the MEnd ligase-DNA complex could assemble with as few as one XLF molecule, and as many as five XLF molecules (Fig. 6*E*).

##### The MEnd Ligase-DNA Complex Has an Anomalously Low Electrophoretic Mobility

MEnd ligase-DNA complexes always had a lower mobility than Ku-XL-DNA complexes (Figs. 1, 2, and 5). The largest fully analyzed Ku-XL-DNA complex contained 5.7 Ku and 7.1 XL molecules (Fig. 4*E*). Based on molecular masses for Ku (150 kDa), XL (185 kDa, from XRCC4 homodimer, 76 kDa, and Ligase IV, 109 kDa), the protein molecular mass of the Ku-XL-DNA complex was 2.2 MDa. The smallest fully analyzed MEnd ligase-DNA complex contained 2.0 Ku, 3.8 XL, and 2.6 XLF (homodimer, 66 kDa) molecules, corresponding to a total molecular mass of 1.1 MDa (Fig. 5*E*). Although the molecular mass of proteins in the MEnd ligase-DNA complex was half the molecular mass of proteins in the Ku-XL-DNA complex, it migrated with a lower mobility in the EMSA. Indeed, the smallest MEnd ligase-DNA complex containing only a single XLF molecule migrated more slowly than the largest Ku-XL-DNA complexes (Fig. 6*A*). Thus, the Ku-XL-DNA complexes and MEnd ligase-DNA complexes exhibited an anomalous difference in mobility.

##### Larger Ku-XL-DNA and MEnd Ligase-DNA Complexes Are Associated with Increased DNA End-joining Efficiency

**FIGURE 7.**
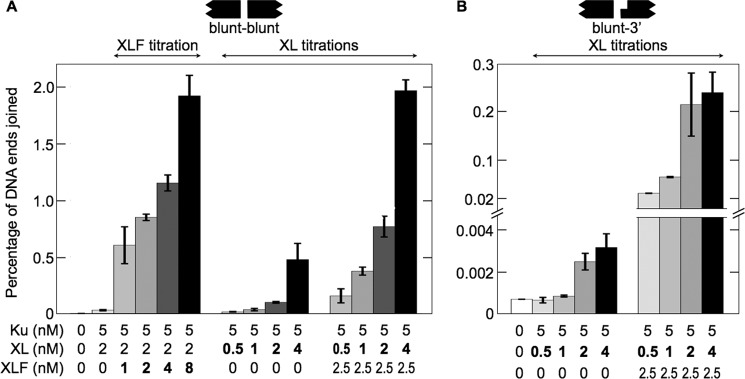
**Increasing concentrations of XL or XLF increase joining efficiency.** Ku and XL with or without XLF were incubated with 800-bp DNA substrates. *Error bars* represent variations in duplicate experiments. *A*, joining of DNA with blunt ends (EcoRV-EcoRV). The data show the effect of increasing XLF and increasing XL concentrations. *B*, joining of DNA with mismatched blunt and 3′ overhanging ends (EcoRV-KpnI). The data show the effect of increasing XL concentrations. The effect of increasing XLF concentrations was reported previously (22).

We measured end joining under conditions favoring intramolecular ligation of a linear DNA substrate. Increasing XLF or XL concentrations led to increased joining of two blunt ends or mismatched blunt and 3′ overhanging ends (Fig. 7). The increased joining efficiency could be due to increased size of the complexes or to increased numbers of complexes. Indeed, increased size and increased number both occurred with increasing XLF or XL concentrations (Figs. 1–6).

## DISCUSSION

Here we show that Ku, XL, and XLF bind DNA cooperatively to form a megadalton protein-DNA complex. The biochemistry recapitulated observations in living cells. We used XRCC4 in a complex with Ligase IV because XRCC4 requires Ligase IV for stable localization to double-strand breaks in living cells (26). In our EMSAs, as in living cells, stable recruitment of XLF to double-strand breaks required Ku, XL, and an intact C-terminal DNA binding domain in XLF (25, 27).

Formation of the MEnd ligase-DNA complex correlated with end joining. The absence of Ku or XL prevented complex formation and as shown previously, abolished end joining (22, 28). Mutant proteins XLF^1–224^ and XLF^L115A^, which disrupted XLF interactions with DNA and with XRCC4, respectively, disrupted complex formation and decreased joining efficiency. Increasing XL and XLF concentrations increased the size of the MEnd ligase-DNA complex and increased end-joining efficiency.

Structural studies show that XRCC4 and XLF can form a helical filament of alternating XRCC4 and XLF molecules (16–19). Depending on crystallization conditions, the helical structure undergoes a full turn of six XRCC4/XLF pairs every 720–860 Å. Because the crystal structures lacked DNA as well as Ku and Ligase IV, they were consistent with several models for how XRCC4 and XLF might bind to DNA.

Several pieces of data indicate that the MEnd ligase-DNA complex contains a filament of alternating XL and XLF molecules bound to the DNA. First, XL and XLF bound to DNA in a 1:1 molar ratio (Fig. 5). Second, XL recruited XLF, and XLF recruited XL (Fig. 6). Third, XRCC4/XLF co-crystals contain filaments of alternating XRCC4 and XLF molecules. Finally, the dimensions of the XRCC4/XLF co-crystal are consistent with the stoichiometry of the largest MEnd ligase-DNA complex, which contained one Ku molecule and five molecules each of XL and XLF bound to 180 bp of DNA (Fig. 5). Because Ku binds 20 bp of DNA (29), one XL/XLF pair would bind about 30 bp of DNA, approximating the distance spanned by the head domains in the XRCC4/XLF co-crystal.

We detected MEnd ligase-DNA containing two Ku molecules, indicating that Ku can interrupt regions of alternating XL and XLF molecules. This complex may form in intact cells, which contain thousands of Ku molecules and only hundreds of XL and XLF molecules (12, 30, 31).

**FIGURE 8.**
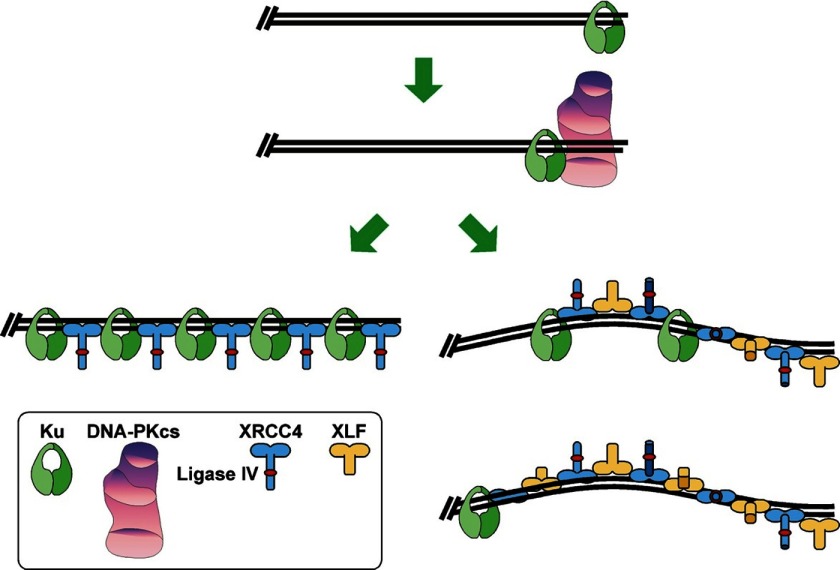
**Model for the Ku-XL-DNA and MEnd ligase-DNA complexes.** Ku binds to the DNA end and slides inward upon recruiting DNA-PKcs (39). DNA-PKcs forms a synaptic complex of two DNA ends and two DNA-PKcs molecules, activating the kinase domain of DNA-PKcs (6). DNA-PKcs autophosphorylation induces a conformational change, releasing the DNA ends for subsequent steps in the NHEJ reaction (28, 40). *Left branch*, Ku-XL-DNA consists of a filament of alternating Ku and XL molecules. *Right branch*, MEnd ligase-DNA contains alternating XL and XLF molecules.

In the absence of XLF, we detected cooperative formation of a Ku-XL-DNA complex. Ku binding to DNA recruited XL, and XL binding recruited Ku. As many as six molecules each of Ku and XL bound to DNA in a 1:1 molar ratio. It is unlikely that the complex contained adjacent Ku molecules because Ku does not interact with itself while bound to DNA (6). Thus, the data suggest that Ku-XL-DNA is a filament of alternating Ku and XL molecules (Fig. 8).

The electrophoretic mobility of a protein-DNA complex generally decreases with increasing protein molecular mass (32, 33). However, interference with normal DNA “reptation” (*i.e.* snake-like movement) through the pores of the gel (34) can decrease mobility further. For example, this occurs with proteins that force DNA into a bent configuration (35, 36).

Surprisingly, the electrophoretic mobility of a 2.2-MDa Ku-XL-DNA complex was greater than a 1.1-MDa MEnd ligase-DNA complex. This could not be explained by synapsis of two MEnd ligase-DNA complexes. None of the many reaction conditions we tested formed a higher mobility MEnd ligase-DNA complex or supported detectable levels of end joining (Figs. 1–6).^3^ Thus, Ku-XL-DNA and MEnd ligase-DNA must form structures with different effects on reptation through the gel. For example, the Ku-XL-DNA may be more flexible than the MEnd ligase-DNA. Alternatively, Ku-XL-DNA may have a smaller radius of gyration than MEnd ligase-DNA.

Cooperative assembly of the protein-DNA filaments suggests that two Ku-XL-DNA, or two MEnd ligase-DNA filaments can undergo end-to-end fusion. Such a structure would allow sliding of the two DNA ends, so that one of the Ligase IV molecules arrayed along the DNA could ligate the ends when they slide into juxtaposition. Ku-XL-DNA, which supports ligation of two DNA ends with cohesive overhangs, may not form a structure that properly aligns DNA ends with mismatched overhangs. By contrast, MEnd ligase-DNA, with its differences from Ku-XL-DNA, both in the molar ratio of XL to Ku and in electrophoretic mobility, must form a distinct structure that successfully aligns mismatched ends.

Cooperative formation of a protein-DNA filament occurs in both nonhomologous end joining and homologous recombination. Our EMSAs showed cooperative formation of Ku-XL-DNA and MEnd ligase-DNA filaments with increasing concentrations of XL or XLF forming progressively larger complexes. Similarly, previously reported EMSAs show cooperative formation of a protein-DNA filament with increasing concentrations of the homologous recombination RecA homolog Dmc1 (37).

Interactions with other NHEJ proteins can occur prior to formation of the full MEnd ligase-DNA complex. In living cells, DNA-PKcs stabilizes the recruitment XRCC4 and/or XL to double-strand breaks (38). For nonligatable ends, DNA-PKcs can recruit the Artemis nuclease to remove damaged terminal nucleotides, and XRCC4 can recruit polynucleotide kinase/phosphatase to prepare terminal 5′-OH or 3′-phosphate groups for ligation (30).

## Footnotes

3 C. Tsai and G. Chu, unpublished data.

## Footnotes

2 The abbreviations used are:

NHEJ: nonhomologous end joining
DNA-PKcs: DNA-dependent protein kinase catalytic subunit
6-FAM: 6-carboxyfluoroscein
MEnd ligase: mismatched end ligase
TAMRA: tetramethylrhodamine
XL: XRCC4/Ligase IV
XLF: XRCC4-like factor.
